# Preoperative risk factors for early recurrence after resection of perihilar cholangiocarcinoma

**DOI:** 10.1093/bjsopen/zrac115

**Published:** 2022-09-20

**Authors:** Ryusei Yamamoto, Teiichi Sugiura, Ryo Ashida, Katsuhisa Ohgi, Mihoko Yamada, Shimpei Otsuka, Takeshi Aramaki, Koiku Asakura, Katsuhiko Uesaka

**Affiliations:** Division of Hepato-Biliary-Pancreatic Surgery, Shizuoka Cancer Center, Shizuoka, Japan; Division of Hepato-Biliary-Pancreatic Surgery, Shizuoka Cancer Center, Shizuoka, Japan; Division of Hepato-Biliary-Pancreatic Surgery, Shizuoka Cancer Center, Shizuoka, Japan; Division of Hepato-Biliary-Pancreatic Surgery, Shizuoka Cancer Center, Shizuoka, Japan; Division of Hepato-Biliary-Pancreatic Surgery, Shizuoka Cancer Center, Shizuoka, Japan; Division of Hepato-Biliary-Pancreatic Surgery, Shizuoka Cancer Center, Shizuoka, Japan; Division of Diagnostic Radiology, Shizuoka Cancer Center, Shizuoka, Japan; Division of Diagnostic Radiology, Shizuoka Cancer Center, Shizuoka, Japan; Division of Hepato-Biliary-Pancreatic Surgery, Shizuoka Cancer Center, Shizuoka, Japan

## Abstract

**Background:**

Early recurrence after curative resection of perihilar cholangiocarcinoma (PHCC) often occurs within a year of surgery. Preoperative predictors of early recurrence remain unclear. The aim of this study was to define reliable preoperative predictors of early recurrence.

**Methods:**

Medical records and preoperative multidetector-row CT of patients with PHCC who underwent resection between 2002 and 2018 were reviewed. Clinical findings, tumour markers, and radiological appearances including a ‘periductal enation sign’ (PES) where there was evidence of soft tissue enhancement appearing to arise from the extrahepatic bile duct, were analysed.

**Results:**

Among 261 patients who underwent resection for PHCC, 67 (25.7 per cent) developed early recurrence. Multivariable analysis identified four preoperative risk factors for early recurrence, namely carbohydrate antigen 19–9 (CA19-9) 37 U/ml or higher (OR 2.19, 95 per cent confidence interval (c.i.) 1.08 to 4.46), positive PES (OR 7.37, 95 per cent c.i. 2.46 to 22.10), mass-forming tumour (OR 4.46, 95 per cent c.i. 1.83 to 10.90), and luminal-occlusion tumour (OR 4.52, 95 per cent c.i. 2.11 to 9.68). The OR of preoperative risk factors were used to define four risk subgroups for early recurrence. The early recurrence rates in the low, moderate, high, and very-high risk groups were 0, 9.4 , 39.7, and 65.0 per cent respectively.

**Conclusion:**

CA19-9, PES, mass-forming tumour, and luminal-occlusion tumour identify patients at higher risk for early recurrence after resection of PHCC.

## Teaser

Carbohydrate antigen 19-9, periductal enation sign, mass-forming tumour, and luminal-occlusion tumour were identified as preoperative risk factors for early recurrence after resection of perihilar cholangiocarcinoma. Risk classification according to these factors was useful for predicting early recurrence.

## Introduction

Surgical resection is the cornerstone to achieve long-term survival in perihilar cholangiocarcinoma (PHCC)^1–3^. Around 24-30 per cent of patients who have undergone resection develop early recurrence within a year of their operation with a dismal prognosis^4–6^. A detailed analysis of preoperative factors that might predict early recurrence has yet to be described, although some studies have reported risk factors for early recurrence after the resection using both preoperative and postoperative factors^5–7^. Recently, the present authors^8^ reported the periductal enation sign (PES) on preoperative multidetector-row CT (MDCT) as being associated with perineural invasion, poor outcomes, and shortened survival in resected distal cholangiocarcinoma. The possible relationship between PES and tumour radiological appearance for predicting perineural invasion and early recurrence in PHCC has not yet been clarified.

The present study aimed to identify preoperative risk factors associated with early recurrence after resection of PHCC.

## Methods

This study was approved by the institutional ethics committee (approval number J2019-142-2019-1-3) and is reported in accordance with the STROBE statement^9^. The medical records and MDCT scans of patients with PHCC who underwent resection with curative intent at Shizuoka Cancer Center between September 2002 and December 2018 were analysed. In-hospital deaths after surgery were excluded. All patients underwent major hepatectomy and bile duct resection with or without vascular resection or pancreatoduodenectomy^10^. The Bismuth classification was used to assess the extent of the tumour^11^. The plasma disappearance rate of indocyanine green clearance (ICGK) and future liver remnant volume were utilized to evaluate the functional reserve of the remnant liver^12^. The ASA physical status (PS)^13^ and Charlson co-morbidity index^14^ were used for preoperative assessments. The preoperative carbohydrate antigen 19-9 (CA19-9) and carcinoembryonic antigen (CEA) values were usually measured within 2 weeks before the day of surgery, after the resolution of jaundice and cholangitis. Neoadjuvant treatment was never performed. Early recurrence was defined as recurrence within 1 year of resection of PHCC^5^.

### MDCT analysis

A MDCT with a standard protocol optimized for cholangiocarcinoma was used for preoperative tumour assessment before biliary stent placement. MDCT was performed in the early arterial, late arterial, portal venous, and delayed phases. Raw data were reconstructed with a slice thickness of 2 mm. MDCT images were reviewed by experienced radiologists blinded to the other clinical findings.

**Fig. 1:**
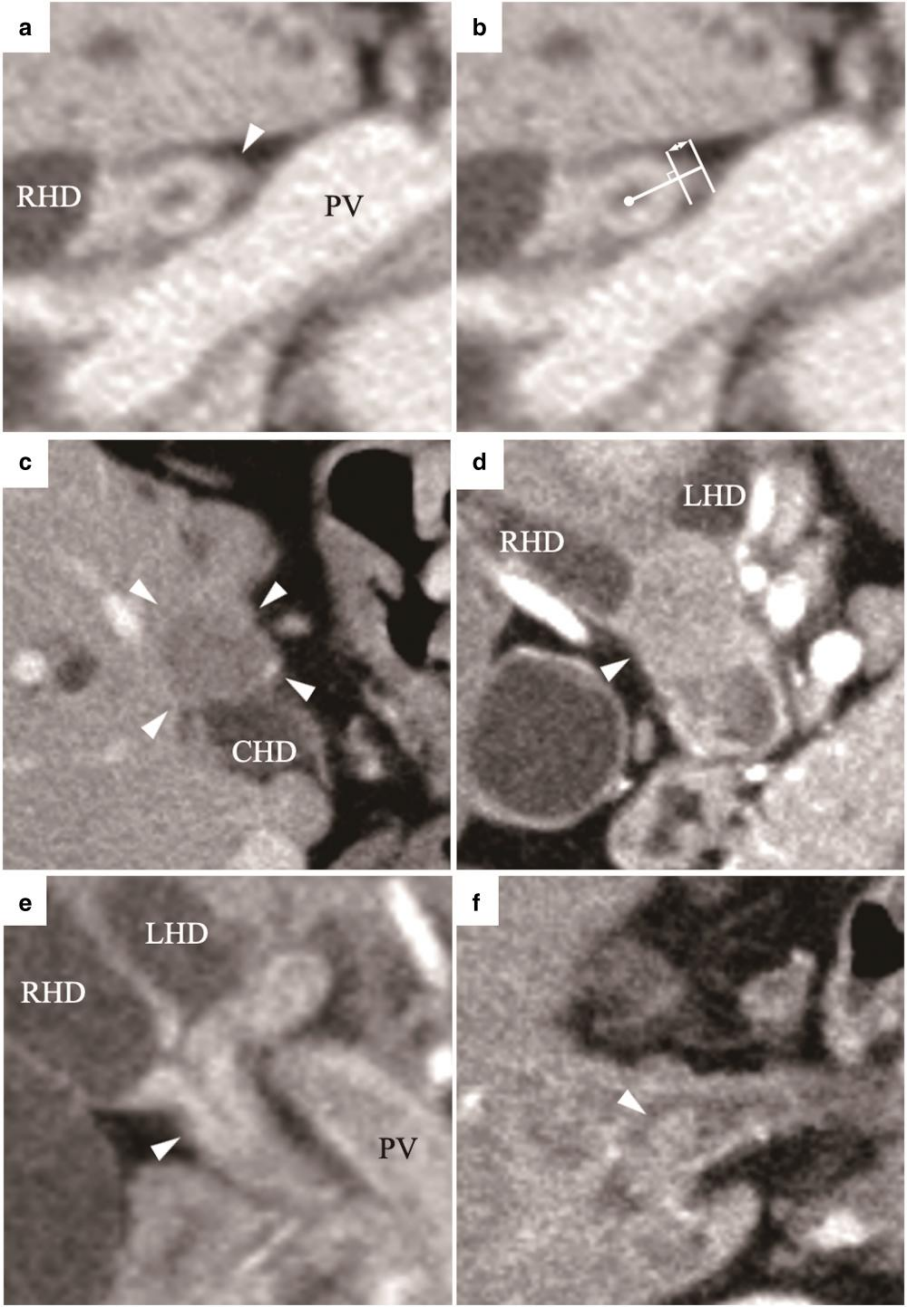
The tumour radiological appearance on multidetector-row CT **a** The arrowhead indicates the PES. **b** The length of the PES was defined as the perpendicular distance from the circumference of the bile duct to the vertex of the enation (arrow). **c** The arrowhead indicates the mass-forming tumour. **d** The arrowhead indicates a luminal-occlusion tumour. **e**,**f** The arrowhead indicates a non-luminal-occlusion tumour. PES, periductal enation sign; PV, portal vein; RHD, right hepatic duct; CHD, common hepatic duct; LHD, left hepatic duct.

As described previously^8^, PES was defined as a surrounding soft tissue enhancement that seemed to emanate from the circumference of the enhanced extrahepatic bile duct at MDCT (*Fig. 1a*). The length of PES was defined as the perpendicular distance from the circumference of the bile duct to the vertex of the enation, and a positive PES was defined as a PES length of 2 mm or more (*Fig. 1b*)^8^. A mass-forming PHCC was defined when an extraductal mass was identified (*Fig. 1c*). Intraductal tumour appearance was divided into ‘luminal occlusion’ where the lumen of the bile duct was completely invisible (*Fig. 1d*) and ‘non-luminal occlusion’ where the lumen remained visible (*Fig. 1e,f*). The tumour abutment angle of the portal vein (PV) or hepatic artery (HA) was assessed, with an angle of 180° or more considered significant. Lymphadenopathy was defined as the detection of enhancing round-shaped lymph nodes with a short diameter of 1 cm or more within the regional lymph node area.

### Postoperative follow-up

Pathological examinations were performed in accordance with the International Union Against Cancer (UICC) TNM Classification eighth edition^15^. Adjuvant treatment was not routinely performed, except for patients who participated in clinical trials (13 patients) and those who had a positive surgical margin at final pathology (18 patients).

Clinical and radiological follow-up was scheduled on a 3-month basis for the first year after resection. Recurrence was diagnosed either through radiological or histological evidence.

### Statistical analyses

Continuous data were described as medians with interquartile ranges and compared using the Mann–Whitney *U* test. Categorical variables were compared using Fisher’s exact test. A logistic regression analysis using stepwise backward selection was performed with multivariable analysis to determine preoperative risk factors for early recurrence after the resection of PHCC. All preoperative variables were entered into the model, and those with a *P* ≥ 0.050 were removed from the final model by backward selection^16^. Factors found to be significant according to multivariable analysis were given weighting points based on their ORs^17^. The factor with the lowest OR was given one point and depending on the ratio of the OR in the other factors to it, two or three points were given. Those points were then summed and divided into four risk subgroups (low, 0–2 points; moderate, 3–4 points; high, 5–6 points; and very-high, 7–8 points). Survival curves were generated using the Kaplan–Meier methods, and differences were compared using the log rank test. Two-sided *P* values <0.050 were considered statistically significant. The statistical analyses were performed using R (version 4.1.0, The R Foundation for Statistical Computing, Vienna, Austria).

## Results

A total of 261 patients underwent curative resection for PHCC and were analysed. Among these, 67 (25.7 per cent) developed recurrence within 1 year of resection. (*Table 1*). Regarding recurrence sites, the liver was the most frequent (46.2 per cent), followed by the peritoneum (31.3 per cent), and lymph nodes (28.4 per cent).

**Table 1.** Site of initial recurrence according to early recurrence

	ER (*n* = 67)	Non-ER (*n* = 194)	*P*
Locoregional	11 (16)	29 (15)	0.844
Distant metastasis*	66 (99)	74 (38)	<0.001
Liver	31 (46)	25 (13)	<0.001
Peritoneum	21 (31)	25 (13)	0.001
Lymph node	19 (28)	18 (9)	<0.001
Lung	11 (16)	18 (9)	0.118
Others	6 (9)	7 (4)	0.103

Values are *n* (%).^*^Including overlap. ER, early recurrence.

*Table 2* shows the clinical and pathological characteristics related to early recurrence. Patients who developed early recurrence had higher CA19-9 and CEA values. According to the MDCT findings, a positive PES, mass-forming tumour, and luminal-occlusion tumour were observed more frequently in the early recurrence group, but the rates of tumour abutment to the PV or HA and lymphadenopathy did not significantly differ.

**Table 2.** Clinicopathologic characteristics according to early recurrence

	ER (*n* = 67)	Non-ER (*n* = 194)	*P*
**Preoperative characteristics**			
Age (years), median (i.q.r.)	70 (64–75)	70 (65–75)	0.872
Sex ratio (M:F)	48:19	132:62	0.647
BMI (kg/m^2^), median (i.q.r.)	21.7 (19.6–23.6)	21.8 (20.0–23.7)	0.613
ASA-PS grade ≥III	11 (16)	20 (10)	0.193
Charlson co-morbidity index, median (i.q.r.)	4 (3–5)	4 (3–5)	0.905
Albumin (g/dl), median (i.q.r.)	3.9 (3.6–4.1)	4.0 (3.6–4.3)	0.124
ICGK, median (i.q.r.)	0.144 (0.137–0.171)	0.152 (0.136–0.175)	0.598
Remnant liver volume (%), median (i.q.r.)	46 (34–70)	47 (39–62)	0.605
CA19-9 (U/ml), median (i.q.r.)	148 (41–792)	47 (18–166)	<0.001
CEA (ng/ml), median (i.q.r.)	3.5 (1.7–5.1)	2.5 (1.5–3.7)	0.025
Biliary drainage	46 (69)	131 (68)	>0.999
Cholangitis	17 (25)	40 (21)	0.493
PV embolization	36 (54)	96 (50)	0.573
Bismuth type, IV	19 (28)	63 (33)	0.647
MDCT findings			
PES-positive	61 (91)	135 (70)	<0.001
Mass-forming	21 (31)	28 (14)	0.004
Luminal occlusion	57 (85)	93 (48)	<0.001
Abutment to PV	32 (48)	81 (42)	0.395
Abutment to HA	38 (57)	107 (55)	0.887
Lymphadenopathy	24 (36)	72 (37)	0.884
**Surgical outcomes**			
Hepatectomy type, right-side	34 (51)	83 (43)	0.319
Combined vascular resection	25 (37)	68 (35)	0.768
Combined pancreatoduodenectomy	10 (15)	40 (21)	0.370
Operating time (min), median (i.q.r.)	578 (477–653)	553 (477–645)	0.587
Blood loss (g), median (i.q.r.)	1447 (966–1986)	1339 (944–1886)	0.326
Blood transfusion	18 (27)	65 (34)	0.363
**Postoperative outcomes**			
Complication, grade ≥3*	27 (40)	87 (45)	0.569
pT3–4†	43 (64)	93 (48)	0.024
pN1–2†	43 (64)	66 (34)	<0.001
pM1†	10 (15)	6 (3)	0.001
Histology, G2–3	51 (76)	111 (57)	0.006
Perineural invasion	56 (84)	144 (74)	0.134
Surgical margin positive	12 (18)	27 (14)	0.431
Adjuvant treatment	6 (9)	25 (13)	0.513

Values are *n* (%) unless otherwise indicated. *According to Clavien–Dindo classification. †According to the UICC 8th edition. ER, early recurrence; ASA-PS, ASA physical status; ICGK, plasma disappearance rate of indocyanine green clearance; CA19-9, carbohydrate antigen 19-9; CEA, carcinoembryonic antigen; MDCT, multidetector-row computed tomography; PES, periductal enation sign; PV, portal vein; HA, hepatic artery.

**Fig. 2:**
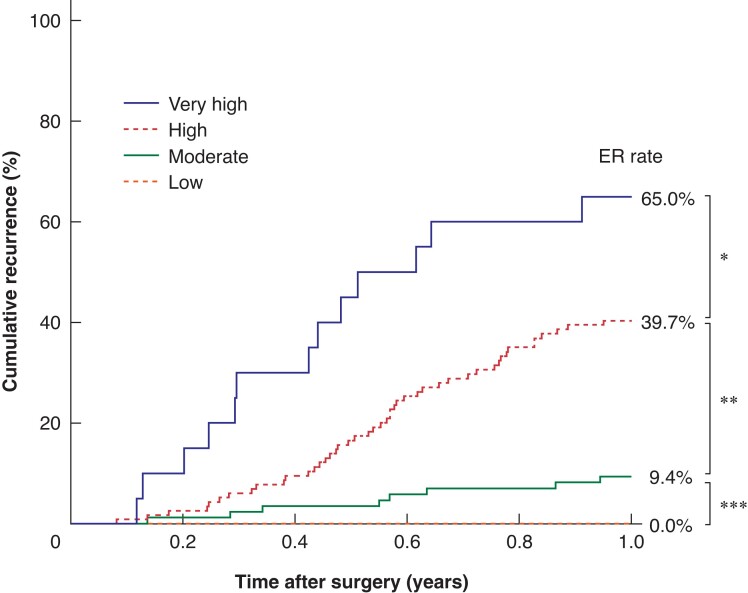
The rate of early recurrence according to the risk classification *P = 0.049, **P < 0.001, ***P = 0.054 (Fisher’s exact test). ER, early recurrence.

Multivariable analysis identified four preoperative risk factors that independently predicted early recurrence: CA19-9 of 37 U/ml or higher (OR 2.19), positive PES (OR 7.37), mass-forming tumour (OR 4.46), and luminal-occlusion tumour (OR 4.52) (*Table 3*). *Figure 2* displays the early recurrence rates ranging from 0 to 65 per cent according to the four risk subgroups created from the multivariable analysis, followed by the association with survival (*Fig. S1*).

**Table 3.** Preoperative risk factors associated with early recurrence

	*n*	ER (%)	OR (95% c.i.)	*P*	Multivariable	
					OR*	*P*
**Age (years)**				0.861	–	–
<75	193	25.4	1.00 (reference)		–	–
≥75	68	26.5	1.06 (0.56–1.98)		–	–
**Sex ratio (M:F)**				0.583	–	–
Female	81	23.5	1.00 (reference)			
Male	180	26.7	1.19 (0.64–2.19)		–	–
**BMI (kg/m^2^)**				0.909	–	–
<25	227	25.6	1.00 (reference)		–	–
≥25	34	26.5	1.05 (0.46–2.38)		–	–
**ASA-PS score**				0.186	–	–
I–II	230	24.6	1.00 (reference)		–	–
III	31	35.5	1.71 (0.77–3.78)		–	–
**Charlson co-morbidity index**				0.996	–	–
<7	248	25.0	1.00 (reference)		–	–
≥7	13	38.5	1.87 (0.59–5.94)		–	–
**Albumin (g/dl)**				0.238	–	–
<4.0	124	29.0	1.40 (9.80–2.44)		–	–
≥4.0	137	22.6	1.00 (reference)		–	–
**ICGK**				0.095	–	–
<0.150	125	30.4	1.61 (0.92–2.82)		–	–
≥0.150	136	21.3	1.00 (reference)		–	–
**Remnant liver volume (%)**				0.815		–
<40	75	26.7	1.08 (0.59–1.98)		–	–
≥40	186	25.3	1.00 (reference)		–	–
**CA19-9 (U/ml)**				<0.001	–	0.030
<37	101	13.9	1.00 (reference)		1.00 (reference)	–
≥37	160	33.1	3.08 (1.60–5.92)		2.19 (1.08–4.46)	–
**CEA (ng/ml)**				0.082	–	–
<5.0	201	23.3	1.00 (reference)		–	–
≥5.0	51	35.3	1.79 (0.93–3.46)		–	–
**Biliary drainage**				0.864	–	–
Absent	84	25.0	1.00 (reference)		–	–
Present	177	26.0	1.05 (0.58–1.91)		–	–
**Cholangitis**				0.418	–	–
Absent	204	24.5	1.00 (reference)		–	–
Present	57	39.8	1.31 (0.68–2.51)		–	–
**PV embolization**				0.549	–	–
Absent	129	24.0	1.00 (reference)		–	–
Present	132	27.3	1.19 (0.68–2.07)		–	–
**Bismuth type**				0.583	–	–
I–III	179	26.8	1.00 (reference)		–	–
IV	82	23.2	1.19 (0.64–2.19)		–	–
**PES**				0.001	–	<0.001
Negative	61	9.2	1.00 (reference)		1.00 (reference)	–
Positive	196	31.1	4.40 (1.82–10.80)		7.37 (2.46–22.10)	–
**Mass formation**				0.003	–	0.001
Absent	212	21.7	1.00 (reference)		1.00 (reference)	–
Present	49	42.9	2.71 (1.41–5.20)		4.46 (1.83–10.90)	–
**Luminal occlusion**				<0.001	–	<0.001
Absent	11	9.0	1.00 (reference)		1.00 (reference)	–
Present	150	38.0	6.19 (2.99–12.80)		4.52 (2.11–9.68)	–
**Abutment to PV**				0.393	–	–
Absent	148	23.6	1.00 (reference)		–	–
Present	113	28.3	1.28 (0.73–2.23)		–	–
**Abutment to HA**				0.824	–	–
Absent	116	25.0	1.00 (reference)		–	–
Present	145	26.2	1.07 (0.61–1.87)		–	–
**Lymphadenopathy**				0.850	–	–
Absent	165	26.1	1.00 (reference)		–	–
Present	96	25.0	0.95 (0.53–1.69)		–	–

ER, early recurrence; ASA-PS, ASA physical status; ICGK, plasma disappearance rate of indocyanine green clearance; CA19-9, carbohydrate antigen 19-9; CEA, carcinoembryonic antigen; PES, periductal enation sign; PV, portal vein; HA, hepatic artery.

Correlation between each risk factor and the pathological features is shown in *Table 4*.

**Table 4.** Pathological features according to carbohydrate antigen 19-9 and multidetector-row CT findings

According to CA19-9			
	CA19-9 ≥ 37 U/ml (*n* = 160)	CA19-9 < 37 U/ml (*n* = 101)	*P*
pT3–4*	92 (58)	44 (44)	0.031
pN1–2*	80 (50)	29 (29)	<0.001
pM1*	13 (8)	3 (3)	0.114
Histology, G2–3	102 (64)	60 (60)	0.514
Perineural invasion	126 (79)	74 (73)	0.368
**According to PES**			
	**PES (+)** **(*n* = 196)**	**PES (–)** **(*n* = 65)**	***P***
pT3–4*	114 (58)	22 (34)	<0.001
pN1–2*	94 (48)	15 (23)	<0.001
pM1*	10 (5)	6 (9)	0.239
Histology, G2–3	124 (63)	38 (59)	0.556
Perineural invasion	186 (95)	14 (22)	<0.001
**According to mass formation**			
	**Mass-forming (+)** **(*n* = 49)**	**Mass-forming (–)** **(*n* = 212)**	***P***
pT3–4*	26 (52)	110 (52)	>0.999
pN1–2*	22 (45)	87 (41)	0.633
pM1*	7 (14)	9 (4)	0.016
Histology, G2–3	43 (88)	119 (56)	<0.001
Perineural invasion	23 (47)	177 (84)	<0.001
**According to luminal occlusion**			
	**Luminal occlusion (+)** **(*n* = 150)**	**Luminal occlusion (–)** **(*n* = 111)**	***P***
pT3–4*	88 (59)	48 (43)	0.017
pN1–2*	74 (49)	35 (32)	0.005
pM1*	8 (5)	8 (7)	0.606
Histology, G2–3	113 (75)	49 (44)	<0.001
Perineural invasion	126 (84)	74 (67)	0.002

Values are *n* (%).*According to the UICC 8th edition. CA19-9, carbohydrate antigen 19-9; PES, periductal enation sign.

## Discussion

Radical surgery represents the cornerstone of PHCC treatment, despite being very invasive, especially once hepatopancreatoduodenectomy and vascular resection are performed^1–3,18–25^. The present study has highlighted the importance of early recurrence after resection for PHCC. Predicting the likelihood of early recurrence before surgery could have a profound effect on decision-making for many patients. The present study identified four preoperative risk factors for early recurrence: CA19-9 of 37 U/ml or higher, positive PES, mass-forming tumour, and luminal-occlusion tumour. CA19-9 is a well established prognostic factor in PHCC^26,27^. The present study revealed that the PES was associated with perineural invasion in PHCC, as shown previously in distal cholangiocarcinoma^8^; however, while a positive PES was an independent risk factor for early recurrence, perineural invasion was not. Luminal-occlusion tumours were associated with the nature of the highly malignant tumour, which might account for the high early recurrence rate.

A mass-forming tumour was also found as an independent risk factor for early recurrence in the present analysis. Large cohort studies of intrahepatic cholangiocarcinoma showed that 22 per cent of patients developed recurrence within 6 months after surgery^28^, and 44 per cent developed recurrence within 1 year^29^. PHCC comprehensively includes intrahepatic cholangiocarcinoma with invasion to the hepatic hilum, as it is difficult to clearly distinguish hilar cholangiocarcinoma and intrahepatic cholangiocarcinoma with invasion to the hepatic hilum on clinical images^15,30,31^. The inclusion of intrahepatic cholangiocarcinoma with mass-forming PHCC may have been responsible for the high early recurrence rate.

To provide a clinically relevant message, a risk classification associated with early recurrence was developed, with increasing rates of recurrence. This identified a group at the highest risk for early recurrence (around 65 per cent) who should be carefully informed about their dismal prognosis before surgery.

The present study has several limitations, including its single-centre and retrospective nature. To validate these results, and in particular, the relatively new concept of PES and luminal occlusion, a multi-institutional study with a large patient population is warranted.

## Supplementary Material

zrac115_Supplementary_Data

## Data Availability

The data sets generated and/or analysed during this study are available from the corresponding author on reasonable request.
