# Erratum to: Preoperative risk factors for early recurrence after resection of perihilar cholangiocarcinoma

**DOI:** 10.1093/bjsopen/zrac143

**Published:** 2022-10-20

This is an erratum to: Ryusei Yamamoto, Teiichi Sugiura, Ryo Ashida, Katsuhisa Ohgi, Mihoko Yamada, Shimpei Otsuka, Takeshi Aramaki, Koiku Asakura, Katsuhiko Uesaka, Preoperative risk factors for early recurrence after resection of perihilar cholangiocarcinoma, *BJS Open*, Volume 6, Issue 5, October 2022, zrac115, https://doi.org/10.1093/bjsopen/zrac115

In the originally published version of this manuscript, the P value in the *Statistical analyses* section was erroneously given as 0.005.

The correct P value is 0.050.

This error has been corrected.

