# Supplementary figures and images for: Preoperative risk factors for early recurrence after resection of perihilar cholangiocarcinoma
Source: BJS Open. 2022 Sep 20;6(5):zrac115. doi: 10.1093/bjsopen/zrac115 (PMC9487653; doi:10.1093/bjsopen/zrac115)

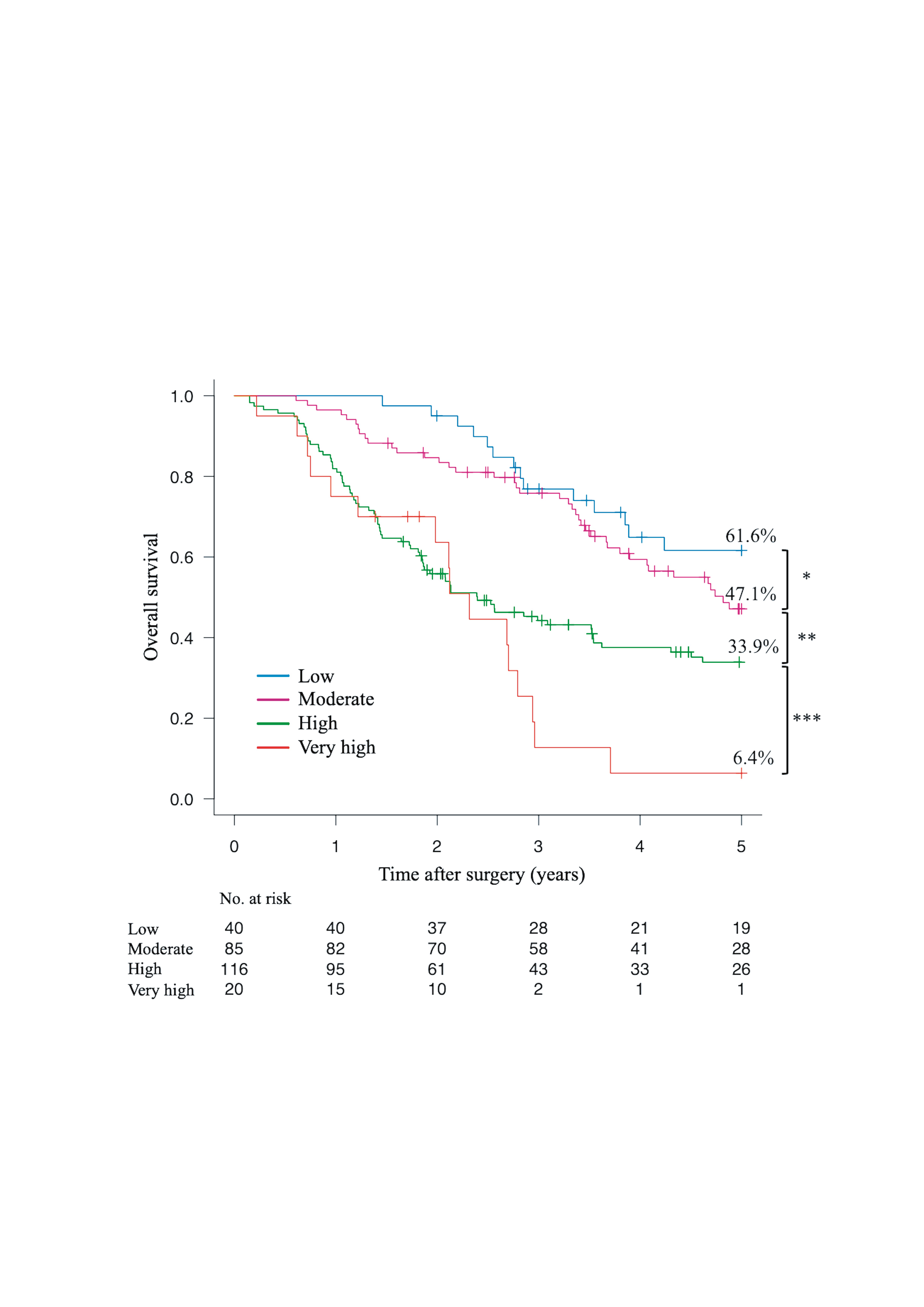

Supplement: zrac115_Supplementary_Data [file zrac115_supplementary_data.jpeg]
